# Complete chloroplast genome sequence and phylogenetic analysis of *Mansoa alliacea*

**DOI:** 10.1080/23802359.2026.2677969

**Published:** 2026-05-24

**Authors:** Yongfang Shi, Liqiang Wang

**Affiliations:** College of Pharmacy, Heze University, Heze City, Shandong Province, P.R. China

**Keywords:** *Mansoa alliacea*, Bignoniaceae, chloroplast genome, phylogenetic analysis, next-generation sequencing

## Abstract

*Mansoa alliacea* (Lam.) A. H. Gentry 1980 is a plant within the family Bignoniaceae, possessing horticultural and medicinal value. Despite its wide range of applications, comprehensive studies on the integrity of its chloroplast genome have been lacking until now. This study presented, for the first time, the sequencing and analysis of the complete chloroplast genome of this plant. The chloroplast genome exhibited a typical quadripartite circular structure, with a length of 163,997 base pairs (bp). It contains 138 genes and has a GC content of 37.65%. Phylogenetic analysis indicates a close relationship between *M. alliacea* and *Anemopaegma* species. These findings provide a robust foundation for future research on the evolution and conservation of *M. alliacea*.

## Introduction

**Figure 1.**
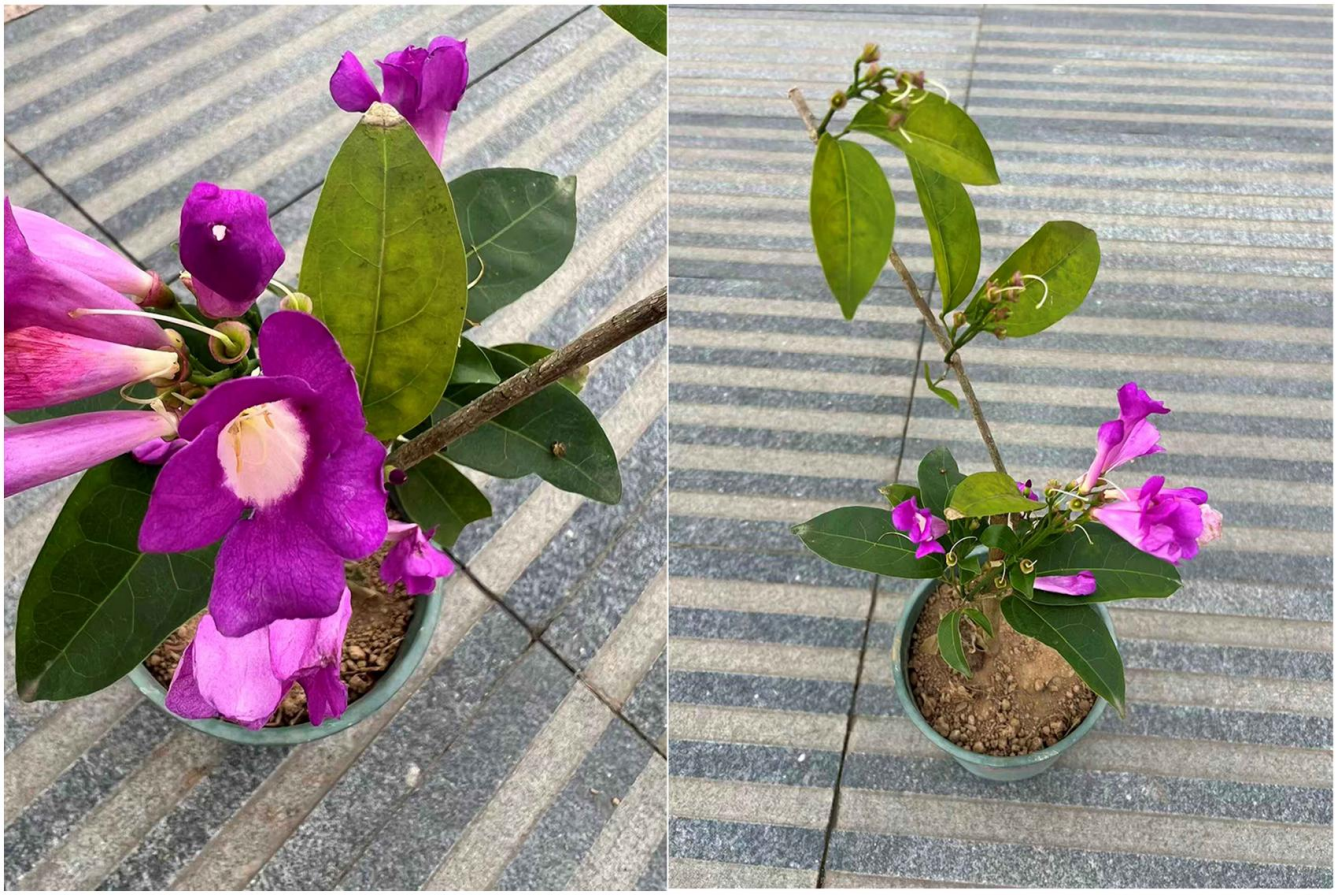
Morphological characteristics of *Mansoa alliacea*. Those photographs were taken by Yongfang Shi at latitude: 35°16′14.11″ N, and longitude: 115°27′36.04″ E. Core identifying features include: Evergreen liana, reaching 3-4 m in length, with pendulous branches and swollen nodes; emits a garlic odor when crushed; opposite compound leaves with 2 leaflets, oblong-ovate, 8-12 cm long, 4-6 cm wide, leathery and glossy, base oblique; terminal leaflet modified into a tendril; cymes axillary and terminal, flowers dense, corolla funnel-shaped, bright purple or purplish-red, turning white when withered.

*Mansoa alliacea* (Lam.) A. H. Gentry 1980 is a plant species belonging to the family Bignoniaceae, native to Guyana and Brazil in South America, and introduced for cultivation in southern China. This plant features dark green foliage and a prolonged flowering period. Its flowers bloom in purple or bluish-purple, fading to a lighter hue later. Crushing fresh flowers or leaves releases a strong garlic-like aroma (Figure 1), contributing to their high horticultural value.

Research in natural product chemistry has identified bioactive compounds in *M. alliacea*, including allicin and alkaloids, among others (Zuñiga-Miranda et al. 2024). The plant exhibits analgesic properties (Valle-Dorado et al. 2022), and its hydroalcoholic extract shows antinociceptive effects in an inflammatory pain model that simulates features of arthritis (Hamann et al. 2019).

Despite its notable horticultural and pharmacological significance, comprehensive studies on the complete chloroplast genome sequence of *M. alliacea* remain limited. This study aims to analyze, sequence, and annotate the complete chloroplast genome sequence of *M. alliacea*. This work is important for further investigations into the genetic diversity of *M. alliacea* and for clarifying its phylogenetic position and evolutionary analysis.

## Materials and methods

Samples of *M. alliacea* were collected from Mudan District, Heze City, Shandong Province, China (Latitude: 35°16′14.11″ N, Longitude: 115°27′36.04″ E) (Figure 1). Voucher specimens are deposited in the Herbarium of Heze University, with the specimen number HZU-2025-1112 (Contact: Liqiang Wang, Email: lys832000@163.com).

Total genomic DNA was extracted and purified from fresh young leaves of *M. alliacea* using a plant genomic DNA extraction kit (Tiangen Biotech Co., Ltd., Beijing, China), followed by quality assessment. After verification of DNA quality, it was fragmented into insert fragments of approximately 200–400 bp for library construction. Sequencing was performed by Wuhan Benagen Technology Co., Ltd. (Wuhan, China) on the Illumina NovaSeq 6000 platform, yielding approximately 48 GB of clean reads (fastq format).

Genome assembly was conducted using GetOrganelle software (v1.7.1) (Jin et al. 2020). Gene annotation was performed using CPGAVAS2 software (Shi et al. 2019), with manual curation of the annotation results *via* Apollo software (Pontius 2018). A circular genome map was generated using CPGview software (Liu et al. 2023). All comparative features in the chloroplast genome—such as IR boundary structure, codon-usage summary, and hotspot/cross-species variability—were visualized using the CPStools software (Huang et al. 2024).

To investigate the phylogenetic position of *M. alliacea*, an additional 42 ingroups from Bignonieae and two outgroups from Tecomeae were selected for phylogenetic analysis. A total of 59 common chloroplast protein-coding genes (PCGs) from the 44 species were aligned using MAFFT (Katoh and Standley 2016). These PCGs, which are universally alignable and free from the alignment ambiguities often present in non-coding regions, were used for phylogenetic analyses. The phylogenetic tree was constructed using IQ-TREE software (v2.0) (Nguyen et al. 2015) based on the multi-alignment results. Bootstrap analysis was performed with 1000 replicates. Based on the Bayesian Information Criterion (BIC) score, the TVM+F + I + R2 model was selected as the optimal nucleotide substitution model.

## Results

**Figure 2.**
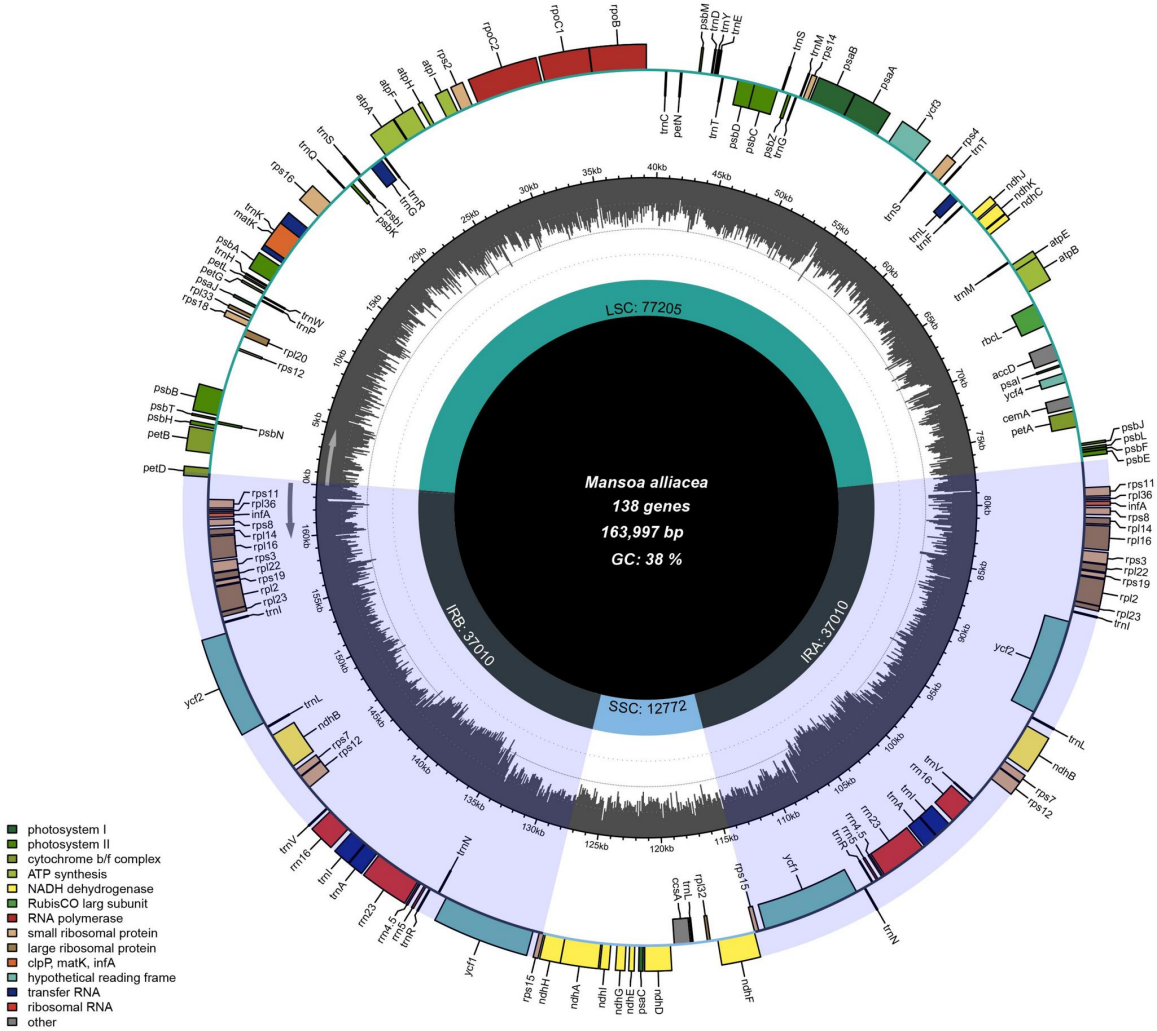
Complete chloroplast genome map of *Mansoa alliacea*. This OGDRAW-generated circular chloroplast genome map illustrates its canonical quadripartite structure: the large single-copy (LSC), small single-copy (SSC), and two inverted repeat (IRa, IRb) regions. Genes are arranged on two concentric rings: the inner ring for clockwise-transcribed genes and the outer for counterclockwise-transcribed genes, with color-coding by functional category. The innermost ring uses a gray bar chart to visualize local GC content, with bar height proportional to its value.

The chloroplast genome of *M. alliacea* exhibits the typical quadripartite structure, with a total length of 163,997 bp. The overall GC content is 37.65%. It consists of a large single-copy (LSC) region of 77,205 bp, a small single-copy (SSC) region of 12,772 bp, and two inverted repeat (IRa and IRb) regions, each 37,010 bp in length (Figure 2). Analysis of sequencing depth for the assembled genome showed an average coverage depth of 8058.58× across all sites, with maximum and minimum coverage depths of 14029× and 43×, respectively (Figure S1).

**Figure 3.**
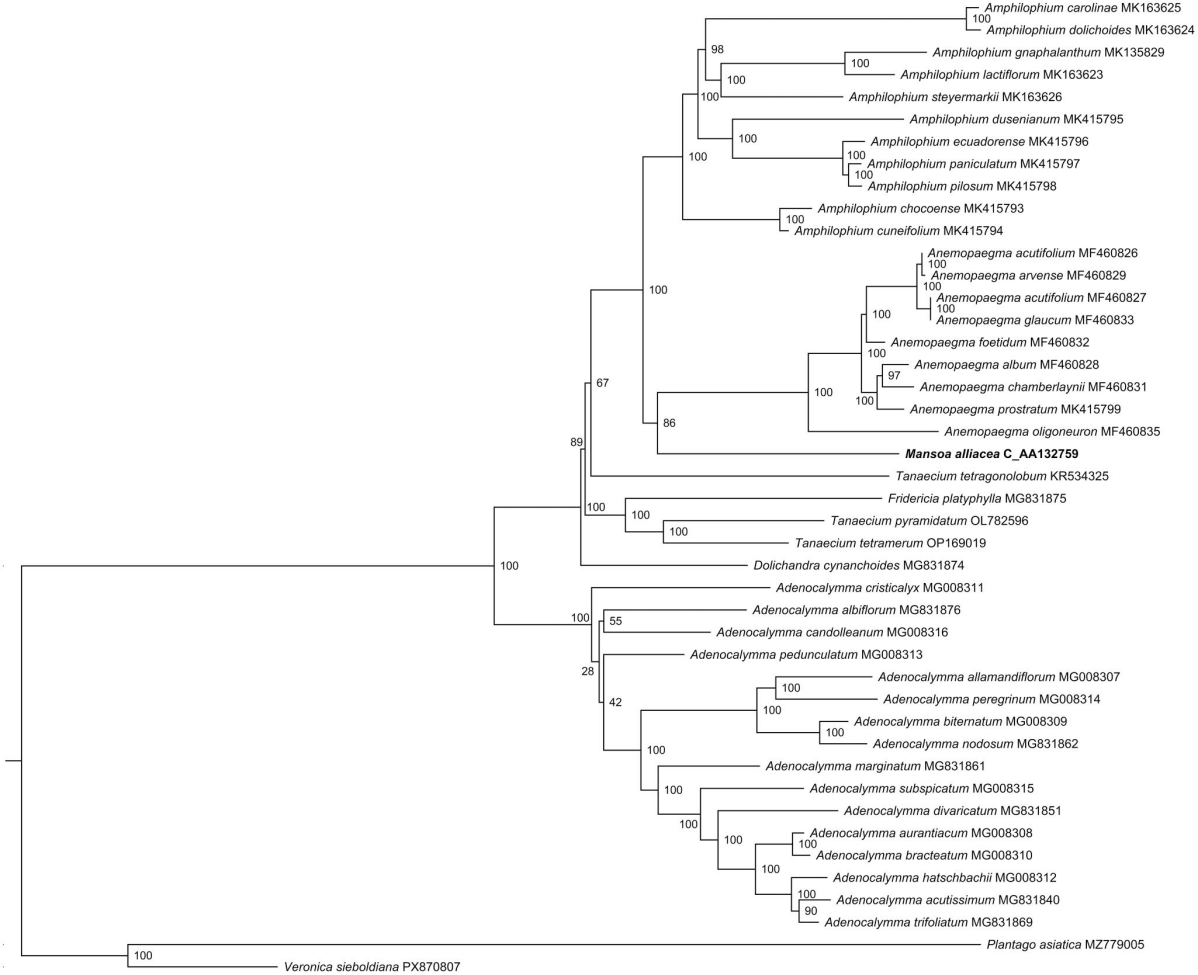
ML Phylogeny of *Mansoa alliacea* and its close relatives. The bootstrap values based on 1000 replicates were shown on each node in the phylogenetic tree. The detailed chloroplast genome citation information was: *Adenocalymma albiflorum* (MG831876) (Fonseca and Lohmann 2018), *adenocalymma candolleanum* (MG008316) (Fonseca and Lohmann 2017), *adenocalymma cristicalyx* (MG008311) (Fonseca and Lohmann 2017), *adenocalymma pedunculatum* (MG008313) (Fonseca and Lohmann 2017), *amphilophium carolinae* (MK163625) (Thode and Lohmann 2019), *amphilophium dolichoides* (MK163624) (Thode and Lohmann 2019), *amphilophium gnaphalanthum* (MK135829) (Thode and Lohmann 2019), *amphilophium lactiflorum* (MK163623) (Thode and Lohmann 2019), *amphilophium steyermarkii* (MK163626) (Thode and Lohmann 2019), *amphilophium dusenianum* (MK415795) (Thode and Lohmann 2019), *amphilophium ecuadorense* (MK415796) (Thode and Lohmann 2019), *amphilophium paniculatum* (MK415797) (Thode and Lohmann 2019), *amphilophium pilosum* (MK415798) (Thode and Lohmann 2019), *amphilophium chocoense* (MK415793) (Thode and Lohmann 2019), *amphilophium cuneifolium* (MK415794) (Thode and Lohmann 2019), *anemopaegma acutifolium* (MF460826) (Firetti et al. 2017), *anemopaegma arvense* (MF460829) (Firetti et al. 2017), *anemopaegma acutifolium* (MF460827) (Firetti et al. 2017), *anemopaegma glaucum* (MF460833) (Firetti et al. 2017), *anemopaegma foetidum* (MF460832) (Firetti et al. 2017), *anemopaegma album* (MF460828) (Firetti et al. 2017), *anemopaegma chamberlaynii* (MF460831) (Firetti et al. 2017), *anemopaegma prostratum* (MK415799) (Thode and Lohmann 2019), *anemopaegma oligoneuron* (MF460835) (Firetti et al. 2017), *mansoa alliacea* (C_AA132759.1, new reported chloroplast genome deposited in CNCB, labeled by bold font), *tanaecium tetragonolobum* (KR534325) (Nazareno et al. 2015), *fridericia platyphylla* (MG831875) (Fonseca and Lohmann 2018), *fridericia trichoclada* (MG831879) (Fonseca and Lohmann 2018), *lundia spruceana* (OQ459817) (Francisco et al. 2023), *tanaecium pyramidatum* (OL782596), *tanaecium tetramerum* (OP169019), *dolichandra cynanchoides* (MG831874) (Fonseca and Lohmann 2018), *plantago asiatica* (MZ779005, outgroup), *veronica sieboldiana* (PX870807, outgroup), *adenocalymma allamandiflorum* (MG008307) (Fonseca and Lohmann 2017), *adenocalymma peregrinum* (MG008314) (Fonseca and Lohmann 2017), *adenocalymma biternatum* (MG00830), *adenocalymma nodosum* (MG831862) (Fonseca and Lohmann 2018), *adenocalymma marginatum* (MG831861) (Fonseca and Lohmann 2018), *adenocalymma subspicatum* (MG008315) (Fonseca and Lohmann 2017), *adenocalymma divaricatum* (MG831851) (Fonseca and Lohmann 2018), *adenocalymma aurantiacum* (MG008308) (Fonseca and Lohmann 2017), *adenocalymma bracteatum* (MG008310) (Fonseca and Lohmann 2017), *adenocalymma hatschbachii* (MG008312) (Fonseca and Lohmann 2017), *adenocalymma trifoliatum* (MG831869) (Fonseca and Lohmann 2018).

This chloroplast genome encodes a total of 138 genes, comprising 94 protein-coding genes (PCGs), 8 ribosomal RNA (rRNA) genes, and 36 transfer RNA (tRNA) genes. Among the annotated genes, 23 genes contain one intron, classified as cis-splicing genes: *ndh*B (2 copies), *ycf*2 (2 copies), *rpl*2 (2 copies), *rpl*16 (2 copies), *rps*11 (2 copies), *pet*B, *rps*16, *atp*F, *rpo*C1, *acc*D, *ndh*A, *trn*A-UGC (2 copies), *trn*I-GAU (2 copies), *trn*K-UUU, *trn*G-UCC, *trn*L-UAA. Three genes contain two introns: *rps*12 (2 copies) and *ycf*3 (Figure S2). Additionally, the *rps*12 gene is a typical trans-splicing gene, with its downstream 3′ end sequences located within the inverted repeat regions, each containing one intron (Figure S3). In the phylogenetic analysis based on common genes, seven genera within the ingroup (Bignonieae) formed a monophyletic clade. Furthermore, *M. alliaceae* constituted a monophyletic group together with *Anemopaegma* genus with high bootstrap values (≥ 86). The topological structure of the phylogenetic tree is consistent with that of the phylogenetic tree constructed based on whole-genome sequences (Figure S4).

To further compare the genome structure of *M. alliacea* with that of related species, the chloroplast genomes of *Adenocalymma* species, which form a monophyletic clade in the phylogenetic analysis, were selected for structural comparison. The *rps*15 occurs as two copies crossing the SSC/IR boundary, consistent with *Adenocalymma*. However, in *M. alliacea*, the LSC/IRb junction is spanned by *pet*D, whereas *pet*B spans this junction in *Adenocalymma* (Figure S5). Hotspot variability analysis revealed polymorphisms in intergenic regions and coding genes, including *rps*11 (π: 0.02104), *rps*18 (π: 0.01525), *ccs*A-*ndh*D (π: 0.04034), and *ndh*D-*psa*C (π: 0.08034) (Figure S6). Across species, RSCU values exhibited variability in codon preference, yet the preferred codon for each amino acid was largely conserved. Minor differences in codon selection for arginine (Arg), serine (Ser), and terminators (Ter) were observed among species (Figure S7).

## Conclusion and discussion

In this study, we reported the first chloroplast genome for *M. alliacea*. The genome was a typical quadripartite molecule with 163,997 in size. The overall GC of the genome was 37.65% and contained 138 annotated genes. Results from the phylogenetic tree show that *M. alliacea* formed a monophyletic clade with *Anemopaegma* species.

Assembly of the *M. alliacea* chloroplast genome showed high similarity in genome length and other characteristics—such as genomic structure, gene count, and GC content—to chloroplast genomes of other species in the Bignoniaceae family (Ma et al. 2020; Xu et al. 2021; Fan et al. 2022). Phylogenetic analysis revealed that all genera formed monophyletic clades, indicating the effectiveness of chloroplast genome sequences for taxonomic classification within the Bignonieae tribe of Bignoniaceae. The congruence between the common gene-based tree and the whole-genome-based tree (Figure S4) echoes the findings of Fonseca and Lohmann (2017) and Francisco et al. (2023), who demonstrated that appropriately selected and edited gene regions can recapitulate genome-level phylogenetic signals in Bignoniaceae. *Mansoa alliacea* clustered into a monophyletic group together with *Anemopaegma* genus, a result consistent with phylogenetic trees constructed from nuclear single-copy gene sequences (Olmstead et al. 2009; Fan et al. 2022; Fonseca et al. 2022).

The findings in this study provided valuable data for research on the genetic diversity and biological characteristics of *M. alliacea*, and offered theoretical support for its further development and application.

## Supplementary Material

FigureS5.jpg

FigureS7.jpg

FigureS4.jpg

FigureS3.png

FigureS6.jpg

FigureS2.png

FigureS1.png

## Data Availability

The complete chloroplast genome sequence of *M. alliacea* in this study was submitted to CNCB-NGDC at https://ngdc.cncb.ac.cn/genbase/ under the accession number C_AA132759.1. The associated BioProject, GSA, and BioSample numbers for raw sequencing data in CNCB-NGDC (https://ngdc.cncb.ac.cn/gsa/) were PRJCA055578, CRR2597441, and SAMC6566916, respectively.
