# Supplementary figures and images for: Complete chloroplast genome sequence and phylogenetic analysis of Mansoa alliacea
Source: Mitochondrial DNA B Resour. 2026 May 24;11(6):786–90. doi: 10.1080/23802359.2026.2677969 (PMC13202697; doi:10.1080/23802359.2026.2677969)

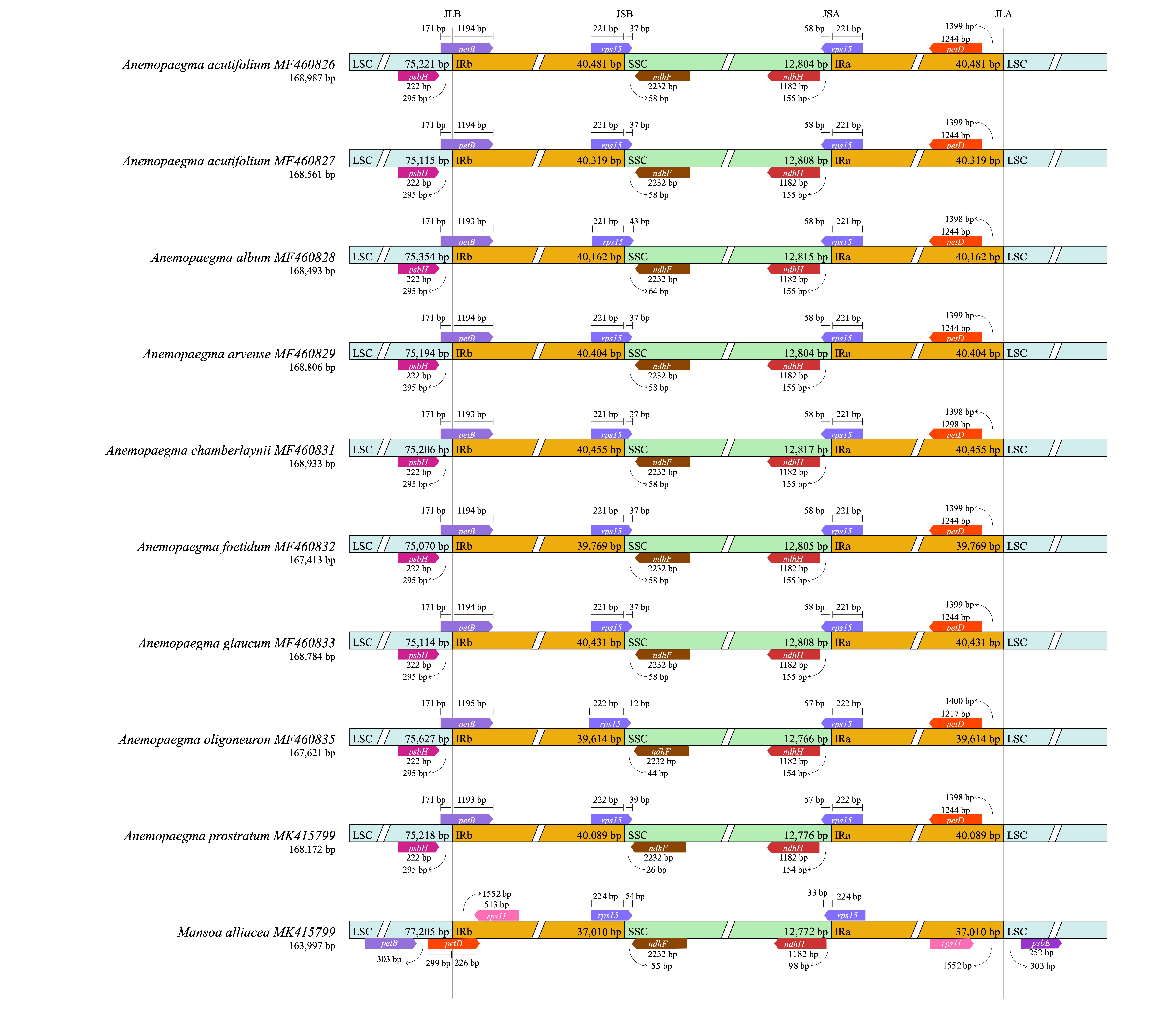

Supplement: FigureS5.jpg [file TMDN_A_2677969_SM6271.jpg]

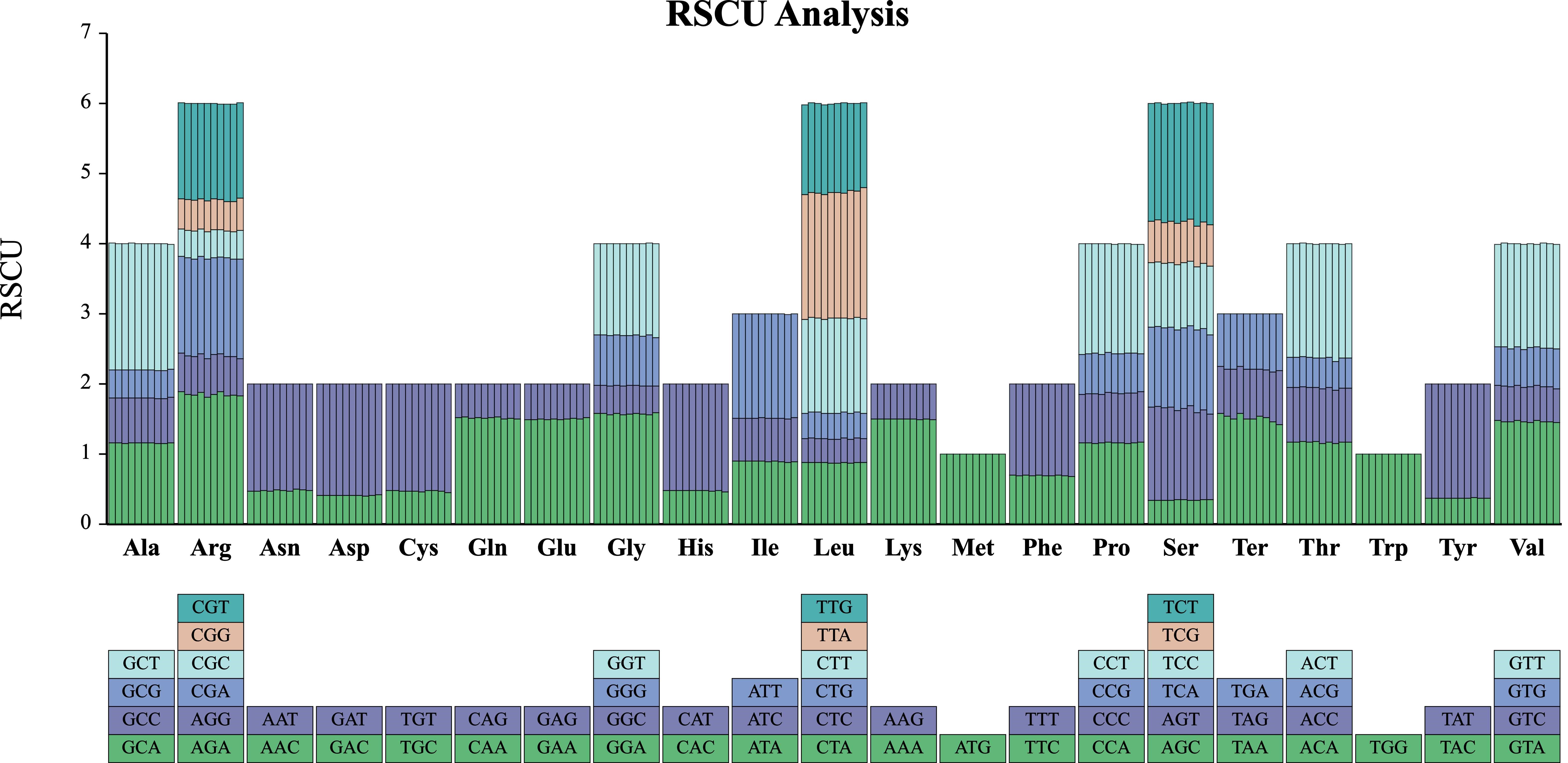

Supplement: FigureS7.jpg [file TMDN_A_2677969_SM6270.jpg]

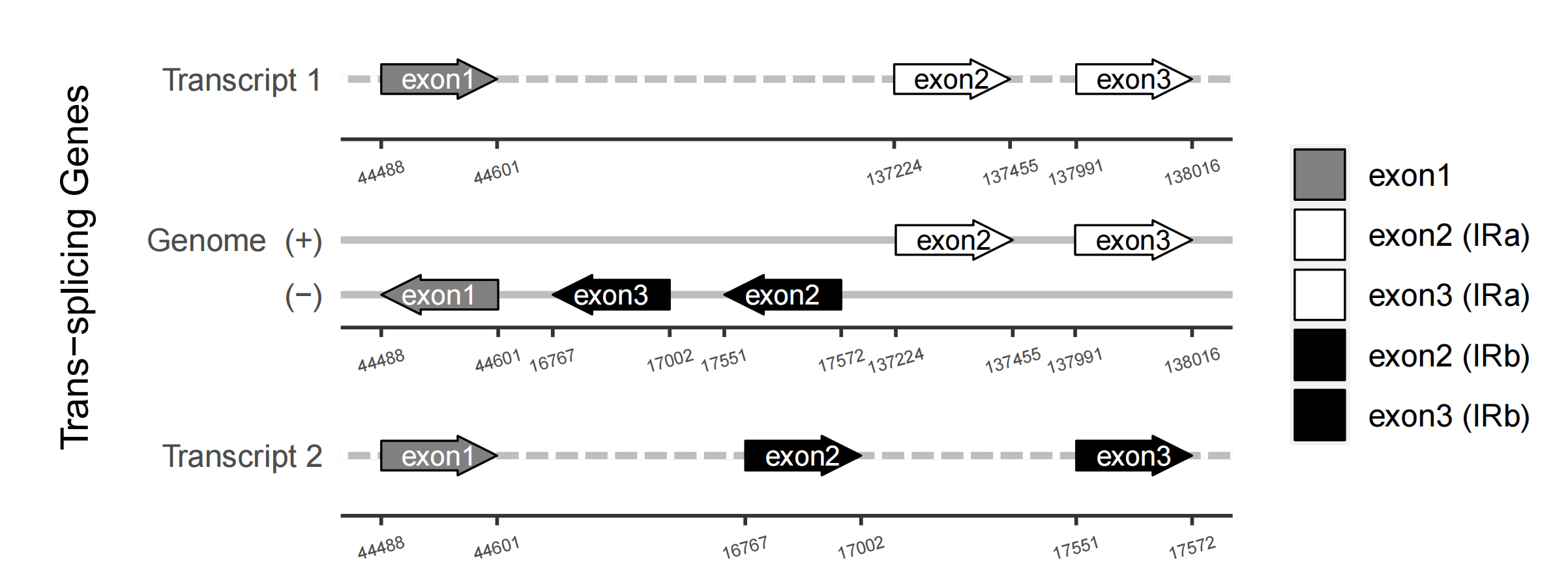

Supplement: FigureS3.png [file TMDN_A_2677969_SM6268.png]

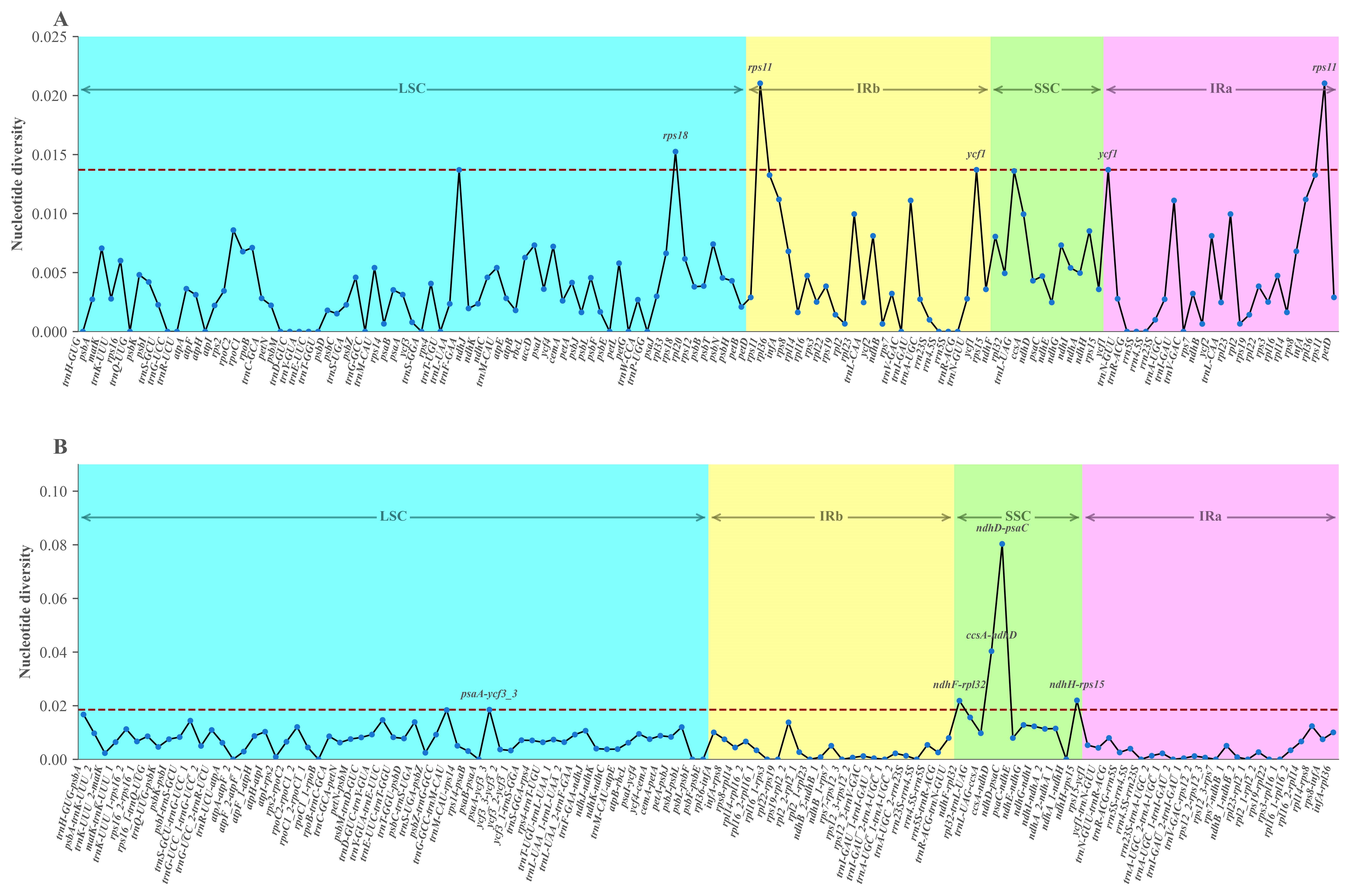

Supplement: FigureS6.jpg [file TMDN_A_2677969_SM6267.jpg]

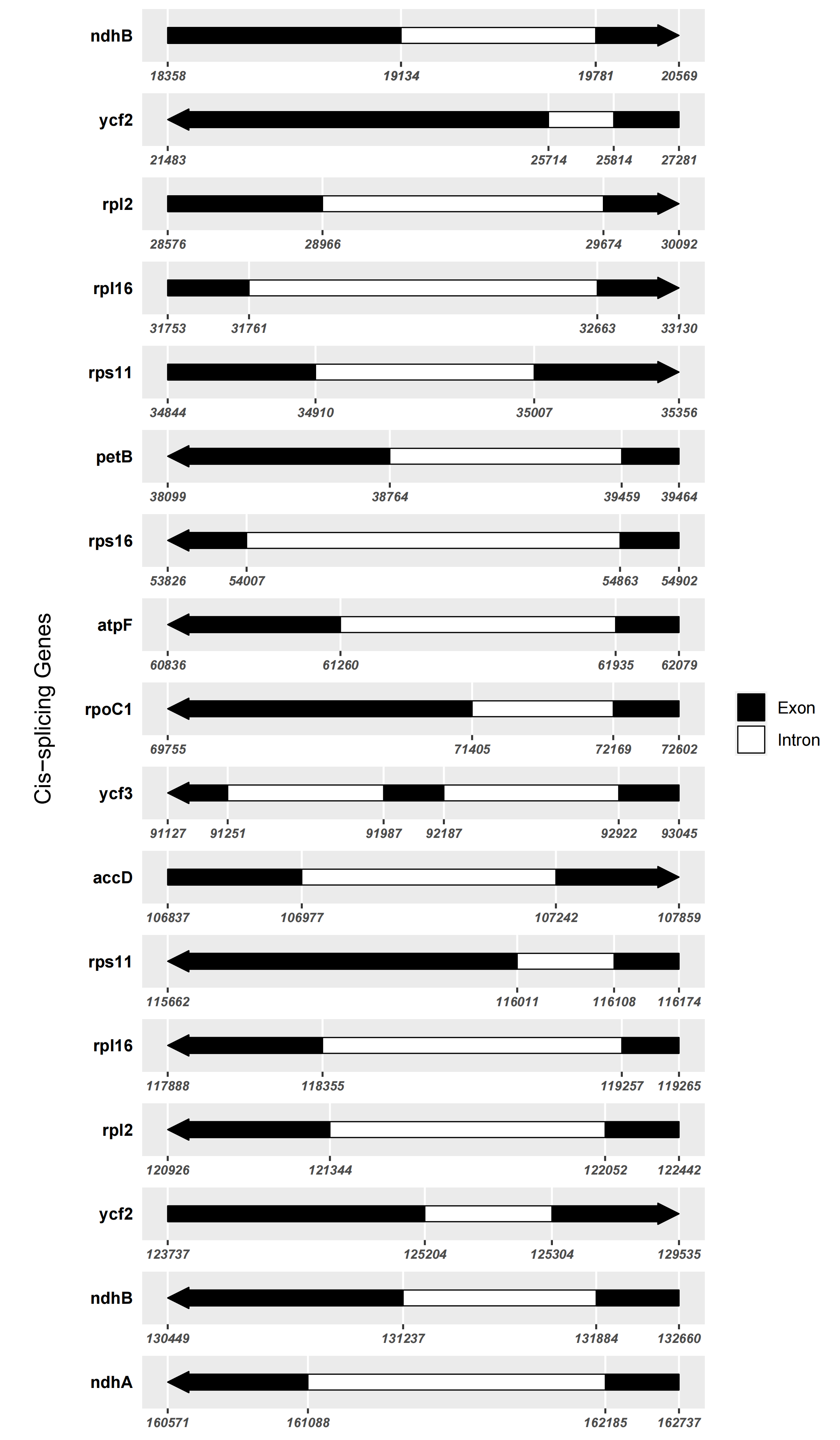

Supplement: FigureS2.png [file TMDN_A_2677969_SM6266.png]

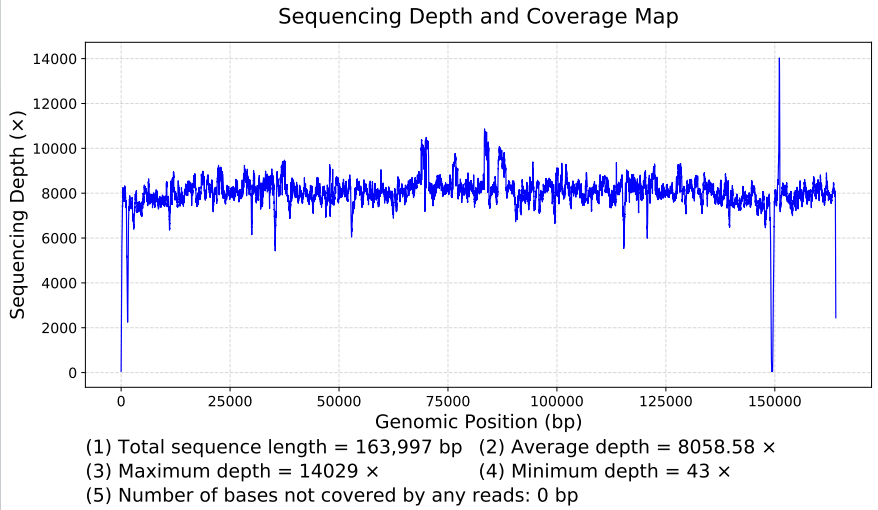

Supplement: FigureS1.png [file TMDN_A_2677969_SM6265.png]
